# Impact of blood flow restriction training timing: Does exercising at dusk or dawn enhance response?

**DOI:** 10.14814/phy2.70644

**Published:** 2025-11-04

**Authors:** L. E. Peskett, A. M. Thomson, B. V. Rioux, D. Nancekievill, J. Arnason, Y. Paudel, M. Sénéchal

**Affiliations:** ^1^ Cardiometabolic Exercise & Lifestyle Laboratory University of New Brunswick Fredericton New Brunswick Canada; ^2^ Faculty of Kinesiology University of New Brunswick Fredericton New Brunswick Canada

**Keywords:** blood flow restriction, exercise, irisin, myokines, time of day

## Abstract

Data suggest that skeletal muscles have an internal clock that dictates training‐related adaptations, which could generate different health benefits in response to exercise timing. However, limited data exist on the impact of blood flow restriction (BFR) training timing on health outcomes. To investigate the impact of 6 weeks of BFR training performed at different times of day on body composition, performance measures, and irisin and PGC1‐α4 expression. Participants (*n* = 31; aged 19–30) who performed 6 weeks of BFR resistance training were categorized into morning (*n* = 16; 05:00–11:00) or afternoon (*n* = 15; 11:00–17:00) groups. A sub‐analysis of responders and non‐responders (top and bottom 25% of muscle strength or lean mass change) was performed. Primary outcomes were changes in body composition, muscle strength, isokinetic measures, and irisin and PGC1‐α4 expression. Time effects were observed for changes in lean mass (*p* < 0.001), relative lean mass (*p* < 0.001), body fat percentage (*p* = 0.02), and all performance measures (*p*s ≤ 0.015). A negative correlation was observed between lean mass change and irisin change (*r* = −0.18; *p* = 0.57). No group or time × group interactions were reported for any outcomes. Six weeks of BFR training provided improvements in body composition and performance outcomes and no changes in irisin and PGC1‐α4 irrespective of timing.

## 1 INTRODUCTION

Current evidence shows that exercising at different times of day can reduce the risk of several chronic conditions. For instance, individuals who exercised in the morning demonstrated a lower risk of cardiovascular disease‐related mortality (Albalak et al., 2023), prostate and breast cancer (Weitzer et al., 2021), and obesity (Chomistek et al., 2016) compared to those who exercised in the afternoon or evening. Ma et al. also reported that participants who accumulated more moderate‐to‐vigorous physical activity in the morning have reduced odds of obesity relative to those active at other times of day (Ma et al., 2023).

A whole body of research has explored how training at different times of day affects performance outcomes. Studies consistently show that repeated sprint intervals performance (Pullinger et al., 2019), time to exhaustion on a cycle ergometer (Kang et al., 2023), and anaerobic power (Knaier et al., 2022) are enhanced when performed in the afternoon or evening rather than the morning. However, evidence regarding strength and power adaptations after training generated mixed results. Krčmárová et al. found that both morning and afternoon training led to significant improvements in strength, with no between‐group differences (Krčmárová et al., 2018). Sedliak et al. observed higher peak torque in the afternoon prior to training (Sedliak et al., 2007). However, this difference disappeared post‐training in individuals who exercised in the morning as there was no longer a significant difference between their morning and afternoon performance (Sedliak et al., 2008). Similarly, morning training has been shown to significantly increase peak power, during a Wingate test, in the morning when compared to the afternoon (Chtourou et al., 2012).

Despite these findings, most studies on exercise timing thus far have focused on traditional forms of exercise training and not considered the impact of timing on key exercise‐induced metabolic regulators. One such alternative modality of exercise, is blood flow restriction (BFR) training. BFR training uses vascular occlusion to induce hypoxia in the working muscles and has been shown to enhance muscle hypertrophy and strength through increased protein synthesis and hormone release (Kelly et al., 2020; Lim & Goh, 2022). The hypoxic environment created by BFR training is also known to induce increased levels of peroxisome proliferator‐activated receptor gamma coactivator 1‐α (PGC1‐α), a transcriptional factor known to follow a diurnal rhythm (Liu et al., 2007). However, these cyclic fluctuations of PGC1‐α have only been demonstrated in rodent models and do not consider PGC1‐α4 expression, an alternatively spliced variant of PGC1‐α associated with increased muscle hypertrophy (Ruas et al., 2012) through the upregulation of insulin‐like growth factor‐1 and irisin (Boström et al., 2012; Zhang et al., 2022), and the downregulation of myostatin (Martínez‐Redondo et al., 2015). Irisin, a myokine cleaved from FNDC5, has browning effects on white adipose tissue (Boström et al., 2012) and contributes to muscle hypertrophy (Huh et al., 2014), suggesting it may have a role in the metabolic benefits of exercise. Yet, no studies currently examine the impact of BFR training at different times of the day on irisin release or PGC1‐α4 expression in humans, which could provide recommendations on the prescription of exercise timing for hypertrophy.

**FIGURE 1:**
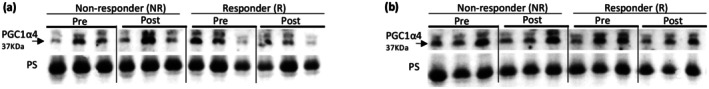
PGC1‐α4 release in morning (a) and afternoon (b) responders and non‐responders as defined by lean mass change.

**FIGURE 2:**
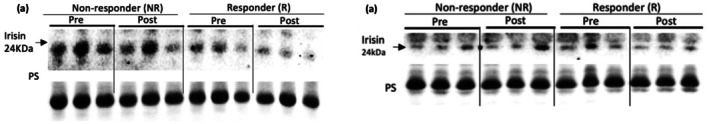
Irisin release in morning (a) and afternoon (b) responders and non‐responders as defined by lean mass change.

**FIGURE 3:**
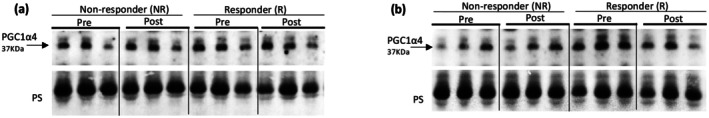
PGC1‐α4 release in morning (a) and afternoon (b) responders and non‐responders as defined by strength change.

**FIGURE 4:**
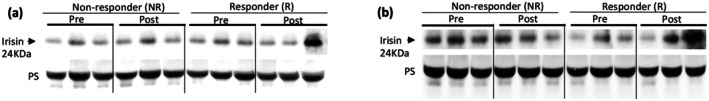
Irisin release in morning (a) and afternoon (b) responders and non‐responders as defined by strength change.

The objective of this study was to determine if BFR training performed at different times of day would have an impact on performance outcomes, body composition, and irisin and PGC1‐α4 expression in young, inactive adults. We hypothesize that there will be a significant difference in the primary outcomes between morning and afternoon groups.

## 2 METHODS

### 2.1 Protocol overview

This is a sub‐analysis of The BFR Study, which was a parallel control experimental study comparing males and females following a 6‐week resistance training intervention in conjunction with BFR (Clinical Trial #: NCT05615831). Participants underwent baseline testing, separated into two visits 1 week apart. Participants began 6 weeks of BFR training within 1 week of the last baseline testing visit. Following the intervention, participants underwent follow‐up testing no later than 1 week following the last exercise session. All testing and exercise sessions took place at the Cardiometabolic Exercise and Lifestyle Laboratory at the University of New Brunswick. The project was reviewed and approved by the University of New Brunswick Research Ethics Board (REB 2021‐124), and all participants provided written informed consent prior to any data collection.

### 2.2 Recruitment and sample

Recruitment was performed between May 2022 and July 2023 through the distribution of promotional flyers, University of New Brunswick's newsletter, posters, social media advertisements through Facebook and Instagram, and promotional booths at local markets.

### 2.3 Inclusion criteria

A total of 31 participants (female *n* = 15), with a mean age of 23 ± 3 years, were included in this sub‐analysis. Participants were included if they were between the ages of 19 and 30 and were physically inactive. Participants were deemed physically inactive if they did not meet the World Health Organization's 2020 physical activity guidelines: 150 min of moderate‐to‐vigorous physical activity (MVPA) and two muscle‐strengthening activities per week. For this study, an average of 10,000 steps/day over a 4–7 day period at baseline was considered to be equivalent to 150 min of MVPA. Participants were eligible if they (1) were aged 19–30, (2) were physically inactive, (3) had no presence of cardiovascular disease such as coronary heart disease, uncontrolled hypertension, peripheral vascular disease, venous thromboembolism, other blood clotting disorders, or hemophilia, (4) did not have surgery, a bone fracture, or a skin graft within the last 3 months, (5) were not pregnant, and (6) completed the study and provided all necessary data for our primary outcomes.

### 2.4 Intervention

Participants took part in 6 weeks of whole‐body BFR resistance training. The intervention consisted of three supervised exercise sessions per week. Each session involved five different exercises (in the following order): chest press, seated row, leg press, knee extension, and knee flexion (seated hamstring curl). The exercise load was individualized to 30% of each participant's 1‐repetition maximum (1‐RM) for each exercise. Participants completed 75 total repetitions broken into four sets for each exercise. The repetition scheme was as follows: set 1: 30 repetitions; set 2: 15 repetitions; set 3: 15 repetitions; set 4: 15 repetitions. This protocol has previously been used in BFR research to induce muscle hypertrophy in a variety of populations and has been suggested in multiple reviews (Kelly et al., 2020; Patterson et al., 2019). At the first exercise session of week four, participants had their 1‐RM reassessed to adjust the 30% 1‐RM exercising loads. Following the 1‐RM reassessment, participants performed two sets per exercise (set 1: 30 repetitions; set 2: 15 repetitions) using the newly adjusted working weight before returning to the original repetition scheme for their remaining sessions.

BFR cuffs were placed at the most proximal portion of the exercising limb (just above the biceps brachii on the arm and near the inguinal crease on the thigh). BFR was achieved using the KAATSU C3 device (KAATSU Global, Inc., Huntington Beach, CA, USA). Cuffs were inflated to 60% of each individual's total limb occlusion pressure. Each participant's total limb occlusion was estimated using equations developed by Loenneke et al. (2015). Cuffs remained inflated during the rest in between the sets of each exercise but were deflated for the rest between each exercise. The resting period between sets was 60 s, and the rest between exercises was 4 min. Occlusion pressure and rest intervals align with what is currently recommended for use for BFR cuffs (Patterson et al., 2019).

### 2.5 Primary exposure variable

#### 2.5.1 Time of day

The research facility was open from 04:00 to 23:00, allowing participants to self‐select their preferred training time within this schedule. Research staff recorded the start time of each participant's exercise session. For this analysis, participants were divided into two categories: morning (05:00–10:59 a.m.) and afternoon (11:00–17:00 p.m.). Group assignment was based on a pre‐established cut‐off of 50%, as used by Qian et al. (2021). As such, if a participant performed >50% of their training sessions within a given time period, they were assigned to that corresponding group. The average time of session for the AM group was 8:55 (95% CI: 8:13, 9:37) with an average adherence to the AM time frame of 84.8 (95% CI: 76.0, 93.5). The average time of session for the PM group was 13:48 (95% CI: 13:01, 14:34) with an average adherence to the PM time frame of 88.8 (95% CI: 82.9, 94.6).

#### 2.5.2 Responders

Participants were categorized as responders and non‐responders for changes in leg muscle strength and lean mass change for each time group. Individuals in the top 25% of the change in leg muscle strength or lean mass were considered responders, while individuals in the bottom 25% of the change in leg muscle strength or lean mass were considered non‐responders.

### 2.6 Primary outcomes

#### 2.6.1 Body composition

Lean mass was estimated using dual‐energy x‐ray absorptiometry (DXA) before and after the six‐week BFR training intervention. Body composition was estimated using a Hologic Horizon® DXA System (Hologic Canada ULC, Mississauga, ON, Canada). Participants presented to the laboratory following a 12‐h fast and were asked to refrain from exercise for a 24‐h period prior to testing. Participants were instructed to wear loose‐fitting clothing with no metal (buckles, zippers, buttons, etc.) and were then instructed to lie supine on the scanner's table and remain still for the duration of the scan. Arms were placed at the participants' sides with palms facing medially and thumbs pointed upwards. For individuals with a width larger than that of the table, they were positioned with one arm outside of the scan area, and the results of the scanned arm were duplicated. The coefficient of variation in our lab for lean mass is 0.6% and for body fat percentage is 0.7%.

#### 2.6.2 Isokinetic measures

Muscular endurance of the dominant knee extensors and flexors was assessed using a HUMAC® NORM™ isokinetic dynamometer system (Computer Sports Medicine, Inc., Stoughton, MA, USA). Prior to testing, participants performed a 5‐min walking warm‐up. The participants were seated and secured to the device using straps across the trunk and thighs. The positioning of the seat was adjusted to the comfort level of the participant, so long as the approximate axis of the knee (through the lateral femoral epicondyle) was aligned with the dynamometer's mechanical axis, and the settings were recorded to be used again following the intervention. Range of motion was then prescribed on an individual basis (0° corresponding to full knee extension). Prior to testing, participants performed a familiarization set of five maximal repetitions at 120°/s. Upon completion of the familiarization, participants were given a 2‐min recovery period before testing commenced. The testing protocol consisted of 30 reciprocal maximal contractions of the knee extensors and flexors performed at 180°/s as previously described (Bosquet et al., 2016). Total work, average power per repetition, and peak torque were recorded.

#### 2.6.3 Muscle strength

Strength was assessed by 1‐RM for the five exercises used during the intervention. 1‐RM was measured during the second baseline testing visit, during the first exercise session of week four of the intervention, and again during the second post‐testing visit. Each participant's 1‐RM was determined using the following protocol: one set of 6–10 repetitions, followed by one set of 3–5 repetitions, followed by small incremental increases for one repetition until failure is achieved within seven attempts. If no failure was achieved within seven attempts, the 1‐RM for that exercise was redone prior to their first exercise session.

#### 2.6.4 Irisin & PGC1‐ α4 measurements

Participants were instructed to fast for at least 12 h prior to blood sample collection. Blood samples were collected before and following the intervention by a registered nurse. Blood was drawn into 3 mL vacutainer collection tubes with an anticoagulant ethylenediaminetetraacetic acid (EDTA). Vacutainers were centrifuged at 2500*g* for 15 min (4°C) and the plasma samples were collected into 1.5 and 2 mL collection tubes inside a biosafety cabinet (Thermo Fisher Scientific, Inc., 1300 Series A2, Marietta, USA) and stored at −80°C until further analysis.

Plasma irisin concentrations were measured, for responders and non‐responders, by gel electrophoresis with western blotting. Plasma samples were diluted 1:24 with double distilled water. 4× Laemmli sample buffer (Bio‐Rad, Cat. #1610747) was prepared (9:1) by adding dithiothreitol (DTT) and added 1:3 into diluted samples. The samples were then boiled at 99°C for 5 min and left on ice to cool down. Gel electrophoresis was performed using two methods: (1) 15 mL of diluted plasma samples was loaded onto 4%–20% mini‐PROTEAN TGX stain‐free gels (Bio‐Rad, Mississauga, Canada, Cat. #4568096) and proteins were transferred to polyvinylidene fluoride (PVDF) membranes (Bio‐Rad, Mississauga, Canada, Cat. #1620177) using the Trans‐Blot Turbo transfer system (Bio‐Rad, Mississauga, Canada, Cat. #1704150). (2) 15 μL of each sample was loaded in each well with Precision Plus Protein™ Kaleidoscope™ Prestained Protein Standards ladder (Bio‐Rad, Cat. #1610375) using a 12% acrylamide resolving gel. Proteins were transferred onto a nitrocellulose membrane (Bio‐Rad, Cat. #1620112) at 90V for 90 min at 4°C using Trans‐Blot Turbo 5× Transfer Buffer (Bio‐Rad, Cat. #10026938). Reversible protein stain (Thermo Scientific, Cat. #24585) was used to visualize the proteins and imaged using ChemiDOC XRS+ (Bio‐Rad).

Membranes were then blocked with every blot blocking buffer (Bio‐Rad, Mississauga, Canada, Cat. #12010020) for 10 min at 60 rpm at room temperature and incubated overnight at 4°C with a rabbit anti‐irisin polyclonal antibody (dilution 1:2000 with blocking buffer) (Adipogen Life Sciences, San Diego, USA, Cat. #AG‐25B‐0027B), or a rabbit anti‐PGC1‐α4 polyclonal antibody (Santa Cruz biotechnology, Cat# SC‐518025) (1:1000 dilution in blocking buffer). 1X Tris Buffer Saline Tween 20 (TBST) was used as the washing buffer (10X‐TBS Cat# 1706435, Bio‐Rad, Mississauga, Canada), diluted with Type 1 water and mixed well with Tween 20 (total concentration 0.05%) (Cat# 1610781, Bio‐Rad, Mississauga, Canada). The membranes were washed 3× for 5 min in TBST and incubated in horseradish peroxidase (HRP) linked anti‐rabbit IgG (CST, MA, USA, Cat. #7074) and anti‐mouse IgG HRP linked (Santa Cruz Biotechnology, USA, Cat. #2357) was used as a secondary antibody (diluted 1:1500 with blocking buffer) and was incubated for 2 h at room temperature. Membranes were washed again 3× for 5 min in TBST. Bands were then visualized with Clarity Max Western ECL substrate (Bio‐Rad, Canada, Cat. #1705062) and densitometric analysis was performed using Image Lab software version 6.1 (Bio‐Rad, Mississauga, Canada).

### 2.7 Statistical analysis

To test for normality within the sample, Shapiro–Wilk tests were performed and confirmed with a visual examination of the data. General characteristics of the sample are presented as mean ± standard deviation or mean (95% CI) for continuous variables and *n* (%) for categorical variables. Differences between the morning and afternoon groups were analyzed using mixed model ANOVA for normally distributed data and using Wilcoxon rank‐sum and Kruskal–Wallis *H* tests for non‐normally distributed data. Data management and statistical analyses were performed using SPSS version 29. A *p* ≤ 0.05 was considered significant.

## 3 RESULTS

Table 1 describes the general characteristics of our sample stratified by morning and afternoon training. No significant differences were observed between the morning and afternoon groups at baseline.

**TABLE 1:** General characteristics of the sample.

	Morning (*n* = 16)	Afternoon (*n* = 15)	*p*‐value
Age (years)	23.3 ± 3.1	22.8 ± 3.0	0.57
Female *n* (%)	8 (50%)	7 (46.7%)	0.86
White *n* (%)	9 (56.2%)	10 (66.7%)	0.46
Steps/day	7145.8 ± 2173.9	5744.70 ± 1802.4	0.06
Weight (kg)	78.0 ± 21.0	80.1 ± 23.2	0.79
Height (cm)	169.3 ± 8.8	172.5 ± 10.5	0.37
BMI (kg/m^2^)	26.95 (23.76, 30.14)	26.95 (22.74, 31.16)	0.63
Waist circumference (cm)	90.8 ± 11.6	92.7 ± 16.6	0.57
Fat mass (kg)	24.85 ± 10.2	26.3 ± 13.6	0.74
Lean mass (kg)	48.9 ± 13.7	49.4 ± 11.6	0.91
Body fat (%)	32.3 ± 8.1	32.2 ± 10.0	0.96
VAT mass (kg)	0.4 ± 0.2	0.5 ± 0.2	0.31
VAT volume (cm^3^)	453.3 ± 166.0	532.1 ± 253.8	0.31
VAT area (cm^2^)	87.0 ± 31.9	102.0 ± 48.6	0.32
RLM (kg/m^2^)	16.8 ± 3.6	16.5 ± 3.2	0.82

*Note*: Data are presented as mean ± standard deviation or mean (95% CI) for continuous variables and *N* (%) for categorical variables. *p*‐value is between‐group difference.

Abbreviations: BMI, Body Mass Index; RLM, relative lean mass; TOD, time of day; VAT, visceral adipose tissue.

Table 2 shows the impact of 6 weeks of BFR training on anthropometrics and body composition. A significant effect of time was observed for change in weight (η_p_^2^ = 0.279; *p* = 0.002), BMI (η_p_^2^ = 0.167; *p* = 0.023), lean mass (η_p_^2^ = 0.394; *p* < 0.001), relative lean mass (η_p_^2^ = 0.39; *p* < 0.001), and body fat percentage (η_p_^2^ = 0.175; *p* = 0.02). No significant group effects or group × time interactions were observed.

**TABLE 2:** The impact of 6 weeks of BFR training on anthropometrics and body composition.

	Morning	Afternoon	*p*‐value		
	Change	Change	Time	Group	Interaction
Weight (kg)	1.65 ± 2.43	0.78 ± 1.46	**0.002**	0.83	0.24
Waist circumference (cm)	0.99 ± 4.87	0.17 ± 2.83	0.43	0.78	0.57
BMI (kg/m^2^)	0.41 ± 0.80	0.20 ± 0.58	**0.02**	0.97	0.42
Fat mass (kg)	0.06 ± 1.05	−0.19 ± 0.94	0.73	0.75	0.49
Lean mass (kg)	1.21 ± 1.50	1.05 ± 1.39	**<0.001**	0.93	0.75
Body fat (%)	−0.38 ± 1.05	−0.49 ± 0.87	**0.02**	0.95	0.75
VAT mass (kg)	−0.01 ± 0.04	0.00 ± 0.04	0.46	0.29	0.74
VAT volume (cm^3^)	−8.19 ± 42.51	−3.33 ± 43.33	0.46	0.29	0.75
VAT area (cm^2^)	−1.56 ± 8.19	−0.50 ± 8.48	0.50	0.29	0.73
Relative lean mass (kg/m^2^)	0.41 ± 0.51	0.34 ± 0.46	**<0.001**	0.80	0.69

*Note*: Data are presented as mean ± standard deviation. *p*‐value < 0.05 is considered significant.

Abbreviations: BMI, Body Mass Index; VAT, visceral adipose tissue.

Table 3 shows the impact of 6 weeks of BFR training on performance outcomes. A significant effect of time was observed for all performance outcomes (all *p*s ≤ 0.015). No significant group effects or group × time interactions were observed.

**TABLE 3:** The impact of 6 weeks of BFR training on performance outcomes.

	Morning	Afternoon	*p*‐value		
	Change	Change	Time	Group	Interaction
Jump power (W)	168.1 ± 207.8	201.6 ± 228.8	**<0.001**	0.71	0.67
Leg press (kg)	12.48 ± 15.05	6.11 ± 22.51	**0.01**	0.89	0.36
Knee extension (kg)^a^	13.65 (9.3, 20.75)	17.70 (9.30, 20.5)	**<0.001**	0.39	‐
Knee flexion (kg)^a^	10.50 (4.63, 16.78)	9.40 (4.65, 20.5)	**<0.001**	0.61	‐
Chest press (kg)	4.49 ± 6.72	8.35 ± 7.82	**<0.001**	0.65	0.15
Seated row (kg)^a^	9.05 (2.30, 13.37)	6.00 (2.30, 13.25)	**<0.001**	0.62	‐
Total work flexors (W)	239.1 ± 369.2	217.0 ± 417.7	**0.003**	0.49	0.876
Total work extensors (W)^a^	416.0 (79.75, 862.75)	361.0 (146.50, 855.50)	**0.002**	0.87	‐
Peak torque flexors (NM)^a^	12.00 (4.50, 17.75)	7.00 (5.00, 15.50)	**0.002**	0.36	‐
Peak torque extensors (NM)	9.81 ± 17.25	6.20 ± 17.08	**0.015**	0.77	0.56
Average power flexor (W)	18.13 ± 20.24	10.27 ± 19.45	**<0.001**	0.58	0.28
Average power extensor (W)^a^	29.50 (1.5, 47.75)	10.00 (3.00, 45.50)	**0.006**	0.17	‐

*Note*: Data are presented as mean ± standard deviation for normally distributed data and as median (IQR) for non‐normally distributed data. *p*‐value ≤ 0.05 is considered signficant.

^a^ Indicates non‐parametric analysis was used.

There was no effect of time, group, or a group × time interaction (*p*s >0.09) for irisin change as defined by lean mass (Figure 2) or strength change (Figure 4) (*p*s >0.27). There was no effect of time, group, or a group × time interaction for either the AM (*p*s >0.47) or PM (*p*s >0.20) group, as defined by lean mass (Figure 1) or strength changes (Figure 3), for PGC1‐α4 (Tables 4 and 5). A significant correlation between irisin change and lean mass change was also observed (*r* = −0.69; *p* = 0.014). PGC1‐α4 change was not correlated with lean mass change (*r* = −0.18; *p* = 0.57). Neither irisin change (*r* = 0.06; *p* = 0.86) nor PGC1‐α4 change (*r* = −0.08; *p* = 0.81) were correlated with strength change.

## 4 DISCUSSION

The objective of this study was to determine if BFR training performed at different times of day would have an impact on body composition, performance outcomes, irisin, and PGC1‐α4 expression. The current study has several important findings relating to exercise timing and BFR training. First, it was found that both morning and afternoon exercisers improved lean mass and relative lean mass and reduced body fat percentage. Second, a significant effect of time was observed for all strength measures with no group effect. Third, only a negative correlation between irisin change and lean mass change was observed. Finally, no change, nor difference, in PGC1‐α4 expression was observed between morning and afternoon responders for lean mass or strength change. These results are important as they shed light on our understanding of exercising at different times of day and changes in body composition. In addition, they provide relevant novel information on the impact of BFR at different times of day and the expression of PGC1‐α4, the master regulator of irisin‐induced muscle hypertrophy.

**TABLE 4:** The impact of response to BFR, as defined by lean mass change, on Irisin and PGC1‐α4 release.

	Morning non‐responder	Morning responder	Afternoon non‐responder	Afternoon responder	*p*‐value		
	Fold change		Fold change		Time	Group	Interaction
Irisin	0.85 ± 0.21	0.83 ± 0.04	1.28 ± 0.46	0.76 ± 0.10	0.29	0.43	0.08
PGC1‐α4	1.03 ± 0.22	0.83 ± 0.13	1.28 ± 0.52	0.96 ± 0.34	0.82	0.87	0.79

*Note*: Data are presented as mean fold change ± standard deviation.

Abbreviation: PGC1‐α4, peroxisome proliferator activator gamma co‐activator alpha 4.

**TABLE 5:** The impact of response to BFR, as defined by strength change, Irisin and PGC1‐α4 release.

	Morning non‐responder	Morning responder	Afternoon non‐responder	Afternoon responder	*p*‐value		
	Fold change		Fold change		Time	Group	Interaction
Irisin	0.85 ± 0.21	0.83 ± 0.04	1.28 ± 0.46	0.76 ± 0.10	0.46	0.39	0.26
PGC1‐α4	1.03 ± 0.22	0.83 ± 0.13	1.28 ± 0.52	0.96 ± 0.34	0.29	0.61	0.26

*Note*: Data are presented as mean fold change ± standard deviation.

Abbreviation: PGC1‐α4, peroxisome proliferator activator gamma co‐activator alpha 4.

### 4.1 Body composition

A significant effect of time for increase in lean mass was observed with no significant group effect or group × time interaction. This increase in lean mass, regardless of training time, is important since low lean mass has been associated with a higher risk of all‐cause mortality (Liu et al., 2022; Spahillari et al., 2016) and cardiovascular mortality (Spahillari et al., 2016). Furthermore, a significant effect of time was observed for a decrease in body fat percentage. However, the decrease in body fat percentage seems to be driven mainly by the increase in lean mass, which has been found in meta‐analyses (Lixandrão et al., 2018; Rodrigo‐Mallorca et al., 2021). Of note, a decrease in body fat percentage with only 6 weeks of BFR training has not been previously reported. However, this needs to be interpreted with caution since some have reported that β2 adrenoceptor signaling could be responsible for muscle hypertrophy with BFR training (Loenneke et al., 2012), which is also known to drive catecholamine‐induced lipolysis of subcutaneous adipocytes (Arner, 1999). Since we do not have the subcutaneous adipose tissue data, this remains a potential hypothesis.

### 4.2 Muscle strength

This study found a significant effect of time for all 1RM measures, with no group effects or group × time interactions. While an increase in strength is expected with an exercise trial, our results are not trivial. Strength has been associated with a reduced risk estimate of all‐cause mortality and has been considered to be a better predictor of all‐cause mortality than lean mass (Newman et al., 2006). Furthermore, muscle strength is also an important risk factor for metabolic syndrome. Sénéchal et al. found that the odds of metabolic syndrome in men aged <50 years were 2.2‐fold greater for those with low composite strength (combination of bench press and leg press 1‐RM) than those with moderate‐high strength (Sénéchal et al., 2014). Therefore, BFR training at any time of the day can be used to induce strength gains and as a preventative health measure.

### 4.3 Isokinetic measures

When looking at isokinetic measures a significant effect of time was observed for all measures, with no group × time interactions or group effects. However, when looking at the lack of group effect this may be due to large variance within groups. Studies have found that BFR training was able to increase peak torque; however, the change observed in our study was not as large as seen with traditional training (Kubo et al., 2006), which also has been reported in the literature (Perera et al., 2022). In addition, studies looking at exercising at different times of day found that both morning and afternoon groups had significant improvements in peak torque and power pre‐ to post‐intervention, with a larger improvement in the morning group (Chtourou et al., 2012; Sedliak et al., 2008), which did not align with our results. A potential reason why we were unable to confirm these findings could be that the studies looking at the impact of the time of day used different testing methodologies to determine peak torque (maximal voluntary contraction) (Sedliak et al., 2008) and peak power (vertical jump) (Chtourou et al., 2012) which indicate that a single output of peak torque and power may be more affected by exercise timing than a repeated output, like what was used in this study.

### 4.4 Irisin

When looking at the response to the intervention, the time of day did not impact the levels of irisin expression in plasma. However, a significant negative correlation between irisin change and lean mass change was observed. This result was interesting, as irisin can increase muscle hypertrophy through the activation of satellite cells (Reza et al., 2017). However, current research does not provide consensus support that exercise‐induced hypertrophy is a result of irisin release. Although a meta‐analysis showed that some studies reported increased irisin following chronic exercise, while others suggest that irisin might remain the same or decrease in longer or supervised exercise trials (Cosio et al., 2021), this is further supported by another meta‐analysis (Qiu et al., 2015). The observed negative correlation between irisin change and lean mass change could be explained by the lack of tissue quantification. It is possible that, by using plasma samples, we may not be able to quantify the increase in irisin associated with muscle hypertrophy as it remains in the muscle to act as an autocrine factor. This hypothesis is supported by other studies that reported an increase in irisin expression in the muscle tissue without an increase in irisin expression in plasma (Archundia‐Herrera et al., 2017). In addition, this result adds to the limited number of studies on irisin and diurnal variation. In fact, one study showed a diurnal variation in irisin with lower irisin levels in the morning and a peak at night‐time (Anastasilakis et al., 2014). Our data add to this literature by showing the impact of chronic BFR training producing a negative correlation between irisin expression and lean mass change, irrespective of training time.

### 4.5 PGC1‐α4

BFR training in rats was found to increase PGC1‐α (Mahdavi et al., 2022), but findings from our study may indicate that BFR may not be effective in increasing the alternatively spliced PGC1‐α4, as there was no change in plasma PGC1‐α4 for responders or non‐responders as defined by lean mass change or strength change. This is counter to what has been found in previous research using traditional training methods (Ruas et al., 2012) and skeletal muscle cell stretching models (Zhang et al., 2022). However, current research on PGC1‐α4 has used muscle biopsies or tissue samples (Ruas et al., 2012), while this study looks at resting plasma levels of PGC1‐α4 pre‐ and post‐intervention. This should lead studies in the future to take both plasma and muscle biopsy measures of PGC1‐α4 to better understand the location of PGC1‐α4 as it induces hypertrophy. Alternative explanations could be that the hypoxic effects of BFR training are not as strong as those used in in vitro studies, which have been used to investigate PGC1‐α expression (Thom et al., 2014). There is also the possibility that the increased lean mass observed in the responders could have been heavily influenced by more well‐understood pathways that induce hypertrophy, such as the Akt/mTOR pathway (Bodine et al., 2001). This is supported by no change in PGC1‐α4 and a negative correlation between irisin change and lean mass change for morning exercisers, as increased levels of irisin have been shown to reduce the function of mTOR (Liu et al., 2018). Research has also shown that ribosomal S6 kinase 1, a regulator of hypertrophy in the Akt/mTOR pathway, was significantly higher after BFR training when compared to performing the same exercises without BFR (Fujita et al., 2007).

### 4.6 Strengths and limitations

This study does have some limitations that need to be acknowledged. First, this study is a sub‐analysis of a larger BFR trial and therefore groups were created following the trial based on participants' exercise times, thereby preventing randomization. Second, the BFR Study had a relatively short duration of only 6 weeks. It is possible some of the differences between groups may have emerged given a longer intervention. Third, testing visit times were not always conducted at the same time as the participant's time of exercise. Finally, the timing of participant food intake prior to or following exercise sessions was not controlled for and could be a confounder. However, the study is strengthened by 100% adherence of participants, as every participant completed every exercise and testing session. The exercise sessions were also supervised by a very small group of staff to keep a controlled environment. Furthermore, using western blotting techniques for irisin analysis allows more accurate results than some other measures, like ELISA, through confirmation of the molecular weight.

## 5 CONCLUSION

This study showed that only 6 weeks of BFR training, performed at different times of day, provided similar changes in body composition and performance measures in young, inactive adults. Irisin expression was negatively correlated with lean mass change as a result of BFR training. Future studies should investigate the relationship between plasma and muscle irisin and PGC1‐α4.

## FUNDING INFORMATION

Public Health Agency of Canada: Healthy Seniors Pilot Project, Project number: C0089.

## CONFLICT OF INTEREST STATEMENT

The authors declare no conflicts of interest.

## ARTIFICIAL INTELLIGENCE GENERATED CONTENT

Artificial intelligence was not used in the creation of this manuscript.
